# Supplementary material for: The causal relationship between the human gut microbiota and pyogenic arthritis: a Mendelian randomization study
Source: Front Cell Infect Microbiol. 2024 Nov 26;14:1452480. doi: 10.3389/fcimb.2024.1452480 (PMC11629706; doi:10.3389/fcimb.2024.1452480)
Supplement: Supplementary file 1 [file DataSheet1.zip › mendelian test/ebi-a-GCST90027808.csv_leave_one_out.pdf]

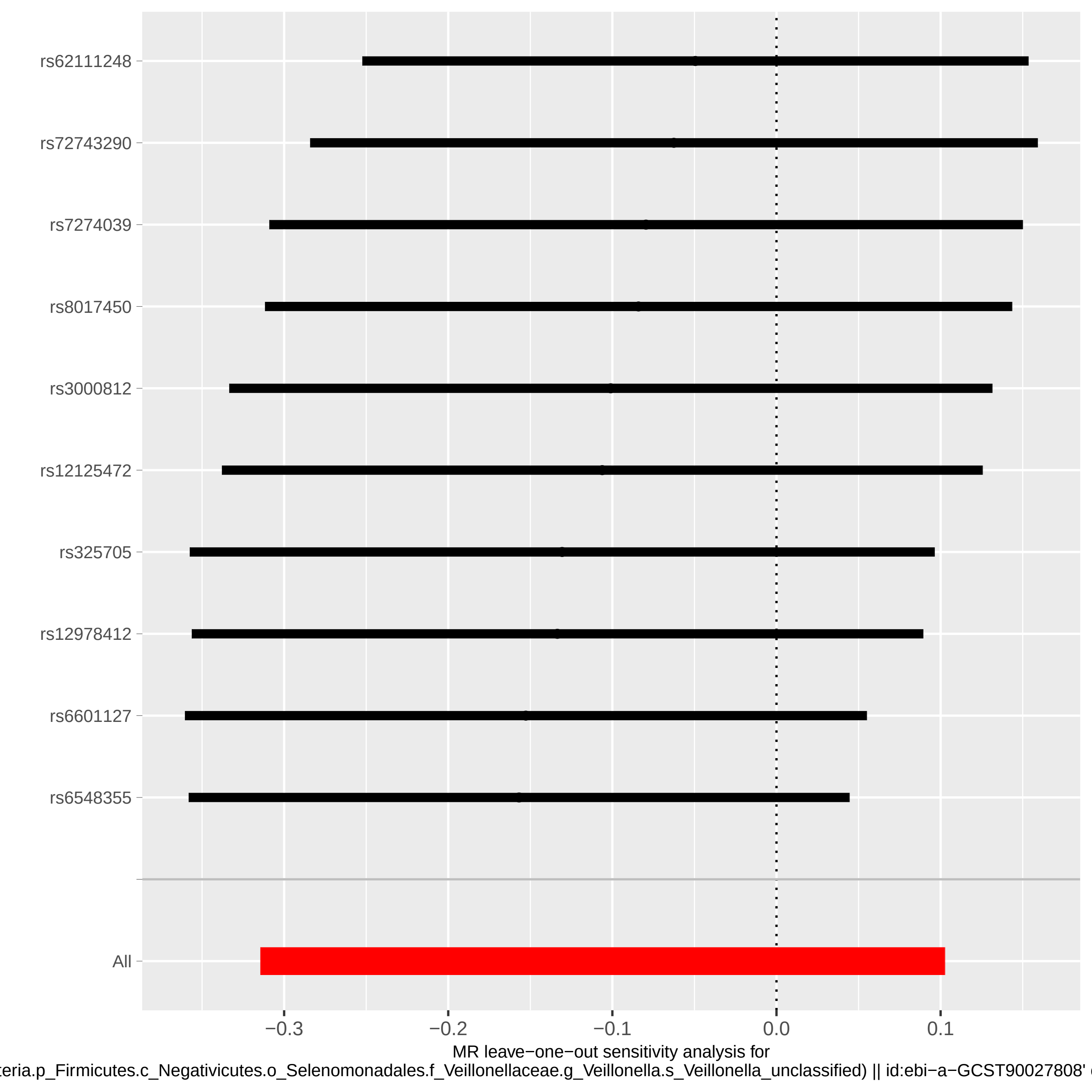

rs62111248
rs72743290
rs7274039
rs8017450
rs3000812
rs12125472
rs325705
rs12978412
rs6601127
rs6548355
All
−0.3
−0.2
−0.1
0.0
0.1
MR leave−one−out sensitivity analysis for
teria.p_Firmicutes.c_Negativicutes.o_Selenomonadales.f_Veillonellaceae.g_Veillonella.s_Veillonella_unclassified) || id:ebi−a−GCST90027808' o
